# Effects of psychosocial interventions among people cared for in emergency departments after a suicide attempt: a systematic review protocol

**DOI:** 10.1186/s13643-021-01609-5

**Published:** 2021-03-05

**Authors:** Ana Paula Coutinho da Silva, Margarida Rangel Henriques, Inês Areal Rothes, Tiago Zortea, José Carlos Santos, Pim Cuijpers

**Affiliations:** 1 Faculty of Psychology and Educational Sciences, University of Porto, Portugal FPCEUP/Center for Psychology at University of Porto CPUP, Rua Alfredo Allen, 4200-392 Porto, Portugal; 2 Department of Clinical Nursing, Health Sciences Center, Federal University of Paraíba, Cidade Universitária, João Pessoa/PB, CEP: 58051-900 Brazil; 3 Suicidal Behaviour Research Laboratory, Institute of Health and Wellbeing, University of Glasgow, Academic Centre, Gartnavel Royal Hospital, 1055 Great Western Road, Glasgow, Scotland G12 0XH UK; 4 Nursing School of Coimbra, Avenida Bissaya Barreto s/n, 3004-011 Coimbra, Portugal; 5 Department of Clinical, Neuro and Developmental Psychology, Amsterdam Public Health Research Institute, Vrije Universiteit Amsterdam, De Boelelaan 1105, 1081 HV Amsterdam, The Netherlands

**Keywords:** Suicide attempt, Emergency department, Psychosocial intervention, Efficacy

## Abstract

**Background:**

The care of the emergency department (ED) for a person after a suicide attempt can act as a protector against future suicidal behavior. For this reason, it is essential that the ED ensure an assistance that involves effective interventions in preventing suicidal behaviors. Among suicidal behaviors, it is known that suicide attempt is one of the most lethal risk factors for consummated suicide. In addition, the risk for further attempts is greater in the period from the immediate post-discharge up to 12 months after the last attempt. This makes the ED a key link in the suicide prevention chain. The purpose of this review is to investigate the effects of psychosocial interventions on suicide prevention, when applied in the ED after a suicide attempt.

**Methods:**

This systematic review protocol was built and registered with the collaboration of a multidisciplinary scientific team. The review will include randomized clinical studies, quasi-experimental trials, and comparative observational studies, all conducted with people (11 years old or more) who have received a psychosocial suicide prevention intervention initiated in the ED after a suicide attempt. The research will be conducted across databases such as Cochrane Library, PubMed, EMBASE, PsycINFO, and DARE. The repetition of a suicide attempt and death by suicide as primary outcomes will be analyzed. The eligibility of the studies and data extraction will be carried out by matched and blind researchers. The risk of bias will be addressed using appropriate instruments. The analyses and synthesis of the results will be both qualitative and quantitative.

**Discussion:**

From a public health point of view, suicide is in itself a public health problem and requires appropriate interventions at different levels of care in order to be prevented. Taking into account that a high percentage of people who died by suicide sought the ED for suicide attempt in the year before their death, the ED is a clinical context with a privileged potential to implement these interventions. Presently, several clinical studies seek to validate interventions to be adopted regarding the prevention of suicidal behavior. Current evidence indicates that different interventions must be strategically combined to reduce suicide attempts and their mortality.

**Systematic review registration:**

PROSPERO registration number CRD42019131040

**Supplementary Information:**

The online version contains supplementary material available at 10.1186/s13643-021-01609-5.

## Background

Suicide is a universal, complex, and multifaceted public health problem. Global statistics estimate that approximately 800,000 people die by suicide every year [1]. The last systematic analysis of the Global Burden of Disease found that suicide is responsible for 1.49% of all causes of death worldwide, the fifteenth cause of death among all ages, and the fifth among those aged between 10 and 24 [2, 3]. Suicide attempt is the strongest predictor of suicidal ideation, subsequent suicide attempt, and death by suicide, and it has been estimated that suicide attempts are twenty times more frequent than suicide [3–8].

Evidence suggests that the risk of repetition of a suicide attempt is greater up to 12 months post-discharge, attaining its maximum risk at immediate post-discharge [3, 9–14]. Moreover, a high percentage of people who died by suicide sought an emergency department following a suicide attempt in the year before their death [15]. Considering this group’s high levels of vulnerability, it has been argued that, when seeking help from a health care facility, people at suicide risk need an empathic response in their first contact as well as a broad psychosocial assessment, discharge under effective planning, and prompt, active, well-coordinated follow-up for months [14].

The contact with the emergency department (ED) may be an opportunity to prevent repetitions of the attempt and death by suicide [16]. In order for EDs to act as a protective factor against suicidal behavior, these services have to guarantee effective interventions. Indeed, interventions initiated in the ED may enable this service a key link in the suicide prevention chain [17–19]. In many cases, the ED was the only health service contact made by those who had attempted suicide [20]. Therefore, EDs should not be undervalued sites to promote suicide prevention; rather, they should be regarded as a useful scenario for initiating the implementation of effective interventions for those who enter the ED due to suicide attempts [17, 20].

The spectrum of mental health intervention encompasses health promotion, prevention, treatment, and maintenance [21, 22]. Clinical studies conducted in EDs have focused their interventions on treatment and maintenance [23–25]. Primary studies have been investigating various psychosocial interventions that can be initiated and implemented in the ED, either individually or associated with each other, such as universal screening for ED-specific suicide risk [23, 26]; individual information sessions [24]; assertive case management interventions [10]; volitional helpsheets—brief psychological interventions [27]; brief contact interventions (BCIs) through emergency or crisis cards (“Green Cards”), phone calls, letters, postcards, or text messages [25, 28–34]; and psychotherapies [35]. In most of these studies, the most commonly analyzed primary outcomes were repeated suicide attempts and suicide after hospital discharge. Other outcomes such as suicidal ideation, social functioning, depression, or hope in life have also been studied, albeit in a more variable proportion [33, 34, 36, 37]. However, such studies do not always yield consensual results [25, 31]. Researchers are becoming increasingly aware that the apparent effects of an intervention can be explained by pre-existing differences in background characteristics between groups and studies should take this variable into account [38, 39].

Among the interventions cited above, studies showed promising results of BCIs when applied shortly after a suicide attempt, but the emphasis is mainly on those whose implementation is in a multimodal program [40]. Overall results from studies involving BCIs, although sometimes heterogeneous, mostly reveal that they may decrease the risk of a repeated suicide attempt [25, 26, 28, 33, 34, 41–44]. Regarding the studies that evaluated the effects of psychotherapies, so far their findings are either not conclusive or lack statistical power [36, 45].

In short, there are studies with promising results [24, 29, 31, 33, 37] but not always consensual [10, 27, 28] and others are even inconclusive [34, 36, 45] which demands the need for systematic reviews. Four recent meta-analyses evaluating the effectiveness of interventions with people who attempted suicide, based on the primary outcomes of repeated suicide attempt and death by suicide, are available [40, 46–48]. Of these, only one systematic review with meta-analysis by Inagaki et al. [46, 48] focused on efficacy studies of interventions specifically initiated in the ED.

The meta-analysis by Inagaki et al. [46, 48] analyzed the effects of all suicide prevention interventions for people who sought the ED due to attempted suicide. The review only included randomized clinical trials (RCTs) of interventions initiated in the ED. With respect to the studied population, considering the inconsistency in the terminology, the authors assume both “suicide attempt” and “self-harm” terms, regardless of the associated suicidal intentionality [32, 49]. The meta-analysis results suggested that active contact and follow-up interventions can be effective in reducing the risk of repeated suicide attempts over a 6- and 12-month period. However, the authors pointed out that the mechanisms leading to those statically significant effects are not clear, possibly due to the existence of differences in interventions and different group backgrounds.

As mentioned, the systematic review by Inagaki et al. [46, 48] points out that the sample RCTs have a substantial degree of heterogeneity of effects and this fact demands caution when looking at the results and when making choices about clinical decisions In addition, addressing different types of interventions and the creation of subgroups by type of intervention leads to a substantial restriction of studies by subgroup, or may occasionally generate false inferences or even inability to infer. Inagaki et al. [46] consider most (more than half) of their RCTs to have a high risk of bias or an obscure risk in five of the evaluation elements of an RCT regarding its methodological quality namely blinding of participants and personnel, blinding of outcome assessment, incomplete outcome data, selective outcome reporting, and other potential sources of bias. And even when evaluating random sequence generation and allocation sequence concealment, the high or obscure risk of bias is over 30%. These assessments, seen as study limitations, suggest that RCTs do not provide sufficient evidence to ignore all non-RCT evidence.

Accordingly, a study by Shrier et al. [50] found that, in some conditions, the inclusion of observational studies increases the accuracy and produces results that are equally or more relevant and valid for what the research aims to answer. Therefore, it becomes appropriate that this Systematic Review be extended to observational studies with comparative analysis.

Additionally, although a growing number of clinical trials are testing different types of suicide prevention interventions, it is not entirely clear which interventions ED professionals can effectively perform in suicide prevention and which subpopulations they can be applied to.

A systematic review of suicide prevention interventions focusing on EDs and displaying a study sample that is both less restrictive as to the methodological design, as well as more restrictive as to the inclusion criteria, only including patients with suicidal intent, can contribute to a better understanding of these interventions and their applicability in the context of EDs.

For this reason, the inclusion criteria for this systematic revision include observational studies with comparative analysis, as well as the suicidal intent as an obligatory criterion for the inclusion of primary studies. Thus, this article’s goal is to describe the protocol of a systematic review which aims at investigating the psychosocial effects of suicide prevention interventions when applied to people who seek the ED after a suicide attempt.

This proposed systematic review is the first part of a study and adds groundwork for its second phase elaborating a suicide prevention guideline in the context of EDs in Portugal.

## Methods

The Cochrane Handbook for Systematic Reviews of Interventions (versions 5.1 and 5.2) was the primary source used to describe the methods of this protocol [51–53]. This protocol was constructed according to the Preferred Reporting Items for Systematic Review and Meta-Analysis Protocols [54] (see Additional file 1) and the Handbook “Meta-analysis in mental health research: A practical guide” [55]. A version of this protocol was registered in PROSPERO [56], with the identification number: CRD42019131040.

### Systematic review question

The research question of the systematic review was built according to the Population (P), Intervention (I), Comparison (C), and Outcomes (O) criteria [57]—among people (11 years old or more) who are admitted to EDs for attempted suicide (P), what are the effects of psychosocial interventions (I) compared to usual/usual improved treatment (C), on repeated suicide attempts and deaths by suicide following psychosocial intervention, adherence to referral for health follow-up, suicidal ideation, psychological symptoms, and social functioning (O)?

### Definition of terms

One of the great challenges in suicide research is the absence of a classification that addresses and clearly and consensually defines suicidal behavior. For Rudd [58], the lack of standard and universal nomenclature is detrimental to studies of intervention efficacy. De Leo et al. [59] further claim that the absence of solid definitions make the task impossible regarding observation of the results of an intervention.

The current study protocol adopts the term “suicide attempt” to reach a more specific population and to remove part of the heterogeneity that a very broad term brings with it. The equivalent term used by the International Statistical Classification of Diseases and Related Health Problems (ICD) is “Intentional Self-harm,” which includes purposely self-inflicted poisoning or injury and suicide attempt [60]. Similarly, in our review, suicide attempt is defined as a self-inflicted injury, including intentional self-poisoning, with a non-fatal outcome for which there is evidence, explicit or implicit, of the intention to die [61].

Regarding the terminology referring to individuals assisted by emergency services, we have adopted the concept of “patients” as designated by the World Health Organization: “every person receiving health care” [62].

### Eligibility criteria

The inclusion criteria were elaborated based on the PICO structure (Population, Intervention, Comparator, and Outcomes) [57]—Table 1. Based on the following inclusion criteria, the articles for the study sample will be selected.

**Table 1:** Inclusion and exclusion criteria in the PICO structure

Population (P)	Intervention (I)	Comparator (C)	Outcomes (O)
**Inclusion criteria**			
Women and men, over 11 years old, of any ethnic group, using emergency departments as a result of an attempted suicide.	All types of psychosocial interventions initiated and/or carried out worldwide in emergency departments after suicide attempts.	Usual treatment and usual improved treatment practiced in each emergency department in which the primary study has been conducted.	Primary: Repetition of suicide attempts and death by suicide. Secondary: Adherence to referral for mental health follow-up, psychological symptoms, suicidal ideation, and social functioning.
**Exclusion criteria**			
Self-harm without suicidal intention (non-suicidal self-injury), people diagnosed with autism, intellectual disability, organic brain syndrome, psychosis, and borderline personality disorder.	Interventions implemented outside the context of emergency services.	–	–
**Study design**	Randomized clinical trials, quasi-experimental trials, and observational studies of controlled case studies and cohort study designs.		
**Language**	Without restriction		
**Year of study**	Without restriction		

### Search strategy

For this systematic review, an initial search on Medline (see Appendix 1), EMBASE, and PsycINFO was adopted and, from these, an adaptation for all other databases. Other databases considered are as follows: Cochrane Library, Cumulative Index to Nursing and Allied Health Literature (CINAHL), Virtual Health Library (BVS), Open Access Theses and Dissertations (OATD), EBSCO Open Dissertations, OpenGrey, National Guideline Clearinghouse, and the Database of Abstracts of Reviews of Effects (DARE). The period of the research is October 2020–February 2021 and will be updated by November 2021.

General search terms were defined based on discussions held by the scientific research team. For this choice, keywords from primary studies and systematic reviews in the studied area were considered. These were as follows: “suicide attempt,” suicide, self-harm, self-poisoning, “emergency department,” and intervention. In addition to these terms, there are also those who identify the study design and specify interventions.

Based on the predefined general terms, all indexed descriptors were searched in all above cited databases. The search strategies were customized for each database, aiming at the specificities of each one, as well as its lexical and taxonomic field of indexed terms, which in turn led to the addition of new terms. The strategies included searches for indexed descriptors and terms present in the titles and abstracts. Sensitivity and precision criteria were taken into account; however, sensitivity was prioritized. The process for the development of strategies was carried out by a researcher under the supervision of a second one, involving consultations with a librarian.

To ensure a wider search and to minimize publication bias, we will expand the preexisting search and include other sources of information. These will be (a) the references cited in the included studies (snowballing technique), (b) citations present in the guidelines of intervention in suicide prevention, (c) suicidology societies (e.g., the Portuguese Society of Suicidology, the International Association for Suicide Prevention, the International Academy of Suicide Research), and (d) experts in the subject. Scientific societies and experts will be contacted by email in order to request any further information or references.

Studies in any language will be included. Although the search terms will be in English, results may be generated in other languages. Google Translator and the Cochrane Task Exchange Platform will be used as an aid to translate articles. Mendeley will be used as a reference manager.

### Inclusion of studies

The software program for the screening of the studies will be Mendeley. For the studies’ eligibility and posteriorly data extraction, a research instrument prepared in Word and Excel and based on the Cochrane Data Collection form Intervention Review - RCTs and non-RCTs [63] is being developed. The first part of this instrument regarding the eligibility of the studies was adapted and subjected to a pilot test to suit the needs of this systematic review (see Additional file 2).

Six researchers will analyze the eligibility of the studies. Three pairs will be formed and in each one the researchers will work independently. This way, data will be simultaneously collected by three groups and in different databases.

This first selection will be made based only on the titles and/or abstracts of the studies read up on the search. The second stage of the studies’ selection will also be conducted by the same researchers in pairs and for each database. In the cases where the full criteria are met, the full text will be examined by both researchers independently. This is the moment when the criterion of suicidal intentionality in cases of self-inflicted injury will be assessed. The studies with a sample of patients who sought the ED for self-inflicted injury and whose intent was suicidal will be included, while studies where doubts about the intentionality arise will be analyzed contacting the authors.

Disagreements over the inclusion of studies will be dealt with together with an external researcher and review consultant. The consultant will be chosen based on the expertise in the area in which the divergence emerged. Measures of formal agreement, kappa statistics, may be used for agreement between reviewers if necessary.

During the eligibility of the studies, a record will be created to justify the reasons why, despite their clinical and methodological relevance, some studies were excluded and also according to which pre-defined inclusion and exclusion criteria.

### Quality appraisal of the evidence

Two investigators will independently evaluate the risk of bias in each included study. After a blind evaluation, a discussion will be carried out between the researchers to identify disagreements, which will then be submitted to an external party for further analysis.

Risk of bias assessments will be conducted using two tools, one for RCT studies and one for non-RCT studies. These are as follows: Revised Cochrane risk-of-bias tool for randomized trials (RoB 2) [64] and Risk Of Bias In Non-randomized Studies - of Interventions (ROBINS-I) [65]. Intra-methodological quality evaluation will be synthesized using tables that will comprise the summary of each study individually, identifying their risks of bias.

### Data extraction

Four researchers, using a database previously built for this purpose, will collect the data. Data extraction will be done in pairs with each researcher working independently. The information to be extracted will be the following: (i) author (s); (ii) date of publication; (iii) country where the research was developed; (iv) study design; (v) characteristics of the population/sample (gender, age, comorbidities, means used to attempt suicide, discrimination between intervention group and control group); (vi) description of the intervention; (vii) description of the comparator; (viii) follow-up period; (ix) outcomes; and (x) result data by outcome (see Tables 2, 3, and 4).

**Table 2:** Data extraction and evaluation

Characteristics of the studies					Effect size	Quality assessment
Characteristics of the studies	Sample characteristics	Characteristics of interventions	Characteristics of comparator	Characteristics of Follow-up	Outcomes	Risk of bias (RCT)
Authors	Gender	Type	Type	Duration	Standard error	Recruitment method
Date of publication/conclusion	Age Socioeconomic status	Professional who implemented it	Professional who implemented it	Detailing	95% CI	Allocation concealment
Journal/source	Comorbidities	Format	Format		*t* test	Blinding of participants and personnel
Country where it was conducted	Use of medicament	Detailing			*p* values	Blinding of outcome assessment
Study design and setting	Means used to attempt suicide					Incomplete outcome data
Primary outcome	Exclusion criteria that were used in the studies					Selective reporting
Secondary outcome						Other sources of bias
						Researcher allegiance
**Quality assessment—risk of bias (non-RCT)**						
Bias due to confounding						
Bias in selection of participants for the study						
Bias in classification of interventions						
Bias due to deviations from intended interventions						
Bias due to missing data						
Bias in measurement of outcomes						
Bias in selection of the reported result						
Overall bias

**Table 3:** Calculation of the effect on continuous data

Studies	Data					
	Outcome	Group	Sample size (***n***) (initial)	Sample size (***n***) (last)	Mean	Standard deviation
**Study 1**	Instrument A	Intervention	–	–	–	–
		Control	–	–	–	–
	Instrument B	Intervention	–	–	–	–
		Control	–	–	–	–

**Table 4:** Calculation of the effect on dichotomous data

Studies	Data			
	Intervention		Control	
	***N***_**success**_	***N***_***total***_	***N***_**success**_	***N***_**total**_
**Study 1**	–	–	–	–
	–	–	–	–
**Study 2**	–	–	–	–
	–	–	–	–

### Grading the body of evidence

The studies will also be evaluated and synthesized according to their outcomes. For each outcome proposed by this review, the quality of the evidence will also be analyzed. The method of this analysis will be the GRADE approach (Grading of Recommendations, Assessment, Development and Evaluation) in order to finally have a summary of the evidence [66].

### Data analysis and synthesis

#### Planned analysis

Eligible sample studies will be reported through a summary of their results. This synthesis will be conducted using a previous classification by type of intervention. This classification of interventions will be conducted according to a process of (a) description of the intervention strategies adopted by each study, (b) qualitative analysis regarding the similarities between interventions. (c) discussion of the data obtained with the scientific team of the systematic review, and (d) categorization of interventions by type/group.

For each categorized type of intervention, their populations and interventions, usual treatments, and results will be described. Randomized clinical studies and observational studies will have separate meta-analyses for appropriate correlations to be made. Through the extracted data, the variability between groups will be presented and evaluated. Measures of effect of interventions by result will be properly reported. This variability will be initially calculated by the *I* ^2^ test and then its origin analyzed for the clinical and methodological aspects of the studies. It is on the basis of this assessment of the variability that subgroups of analysis will be formed. The aggregation of measures of effect will be performed in studies or subgroups whose data—participants, interventions, and outcomes—are sufficiently similar using a random effects model. In the case of those studies that have multiple results to measure the same construct, the option will be to group all instruments within the study so that an effect size is obtained and thus used in the grouping between studies.

Continuous data will be presented according to their weighted mean differences, with 95% confidence intervals (CIs). Dichotomic data will be presented according to relative risk also with CIs of 95%. Statistical significance will be indicated by a *p* < 0.05.

The R package, Metafor, will be used for all statistical synthesis of the evidence.

Regarding the studies considered outliers that cause great variability and those that do not obtain sufficient data to group them, these will only be included for descriptive analysis purposes.

#### Subgroup and sensitivity analysis

Subgroup analysis and, where applicable, meta-regression analysis will be performed for features that are apparently sources of heterogeneity. This study assumes that there will be at least subgroups by classificatory type of interventions, since it would not be accurate to combine different types of interventions in the same analysis, even though the same construct is being measured. Other factors such as age, follow-up time, characteristics of the intervention, and the comparator may also be criteria for subgroup analysis.

A heterogeneity with *I* ^2^ > 50% will be investigated through sensitivity analysis. According to the sensitivity analysis, further decisions will have to be made about study bias risk, age limits adopted for participants, and use of different measures between and intra-studies to examine the effects of the same construct. Publication bias will be analyzed using a funnel chart.

## Discussion

It is well known and consensual that attempted suicide is among the strongest predictors for suicide and general cause of death [13]. People who attempt suicide need immediate treatment, since it was found that the risk of a new attempt is greater in the immediate post-discharge period up to 12 months after the previous attempt [11, 13]. Therefore, initiating an intervention in the ED, as well as maintaining mental health care after discharge, acts as protectors in preventing suicide [16].

An ED with quality of care for people who attempt suicide is built on evidence-based recommendations. Currently, there are several intervention studies that can support these recommendations for preventing suicidal behavior [10, 26, 27, 31, 34, 37, 41, 42]. However, the results of these studies are not always consistent with each other when examining efficacy. Increasingly, researchers have become aware that the apparent effects of an intervention can be explained by preexisting differences in background characteristics between groups, and there is a need for studies that make an analysis based on specific populations [38, 39]. It is also true that among many studied interventions, there is none that is clearly more efficient than others and that only strategic combinations can provide the best results [39].

This review may become limited due to its restriction to people who attempted suicide. Even so, we consider this a viable way to make an analysis with less differences in background characteristics. A high degree of heterogeneity between studies is also likely to be found; therefore, in response, we initially planned subgroup analyzes. The objective is that this systematic review with meta-analysis generates propositions to current practices.

This systematic review has possible and predicted limitations due to the elaboration of its protocol. The inconsistency of terms in suicidology is a limiting factor regarding the search for articles and the subsequent eligibility of studies. Also, due to all the complexity and the multifactorial aspects of suicidal behavior and its treatment, we are aware and do not wish to underestimate the heterogeneity arising from factors such as aspects involving suicidal intentionality and associated risk factors accumulated in the populations of clinical studies. In addition, it is important to highlight the possible relevant differences between the usual treatment used in different studies. The effort will be to carry out a careful review of the heterogeneity between studies, in order to minimize the inconsistencies that may constitute a total impediment to decision making.

The current registration of the protocol for this review at PROSPERO may undergo changes, provided that they are approved by all authors. Any changes to the protocol will be explained and described in the final manuscript of this systematic review.

## Supplementary Information

**Additional file 1.** PRISMA-P 2015 Checklist.

**Additional file 2.** Cochrane data collection form.

## Data Availability

The data extracted and analyzed in this systematic review will be available with the corresponding author under justified request.

## Appendix

### Appendix: Proposed search syntax for MEDLINE, using PubMed

Suicide Attempt

1. "Suicide Attempt"[Title/Abstract]

2."Suicide Attempted"[Title/Abstract]

3."Attempted suicide"[Title/Abstract]

4. parasuicide[Title/Abstract]

5. parasuicides[Title/Abstract]

6. selfharm[Title/Abstract]

7. sel-fharm[Title/Abstract]

8. selfharming[Title/Abstract]

9. self-harming[Title/Abstract]

10. "deliberate self-harm”[Title/Abstract]

11. "deliberate selfharm”[Title/Abstract]

12. Self-Injurious Behavior[MeSH Terms]

13. “Behavior, Self-Injury”[Title/Abstract]

14. "Self-Injury Behavior"[Title/Abstract]

15. "Self-Injurious Behaviors"[Title/Abstract]

16. "Self-Injurious Behavior"[Title/Abstract]

17. Self-Injury[Title/Abstract]

18. Self-Injuries[Title/Abstract]

19. "Self-Destructive Behavior"[Title/Abstract]

20. "Behavior, Self-Destructive"[Title/Abstract]

21. "Behaviors, Self-Destructive"[Title/Abstract]

22. “Self-Destructive Behavior”[Title/Abstract]

23. “Self-Destructive Behaviors”[Title/Abstract]

24. “Deliberate Self-Harm”[Title/Abstract]

25. “Self-Harm, Deliberate”[Title/Abstract]

26. "Deliberate Selfharm"[Title/Abstract]

27. Self-poisoning[Title/Abstract]

28. “deliberate self-poisoning”[Title/Abstract]

29. “deliberate selfpoisoning”[Title/Abstract]

30. Selfpoisoning[Title/Abstract]

31. “intentional self-poisoning”[Title/Abstract]

32. suicide[MeSH Terms]

33. suicide[Title/Abstract])

34. Suicide, Completed[MeSH Terms]

35. “Suicide, Completed”[Title/Abstract]

36. OR “Completed Suicides”[Title/Abstract]

37. “Suicides, Completed”[Title/Abstract]

38. “Completed Suicide”[Title/Abstract]

39. (1 to 38, OR)

Psychosocial Intervention

40. Crisis Intervention[MeSH Terms]

41. "Crisis Intervention"[Title/Abstract]

42. "Crisis Interventions”[Title/Abstract]

43. “Intervention, Crisis”[Title/Abstract]

44. “Interventions, Crisis”[Title/Abstract]

45. Interventions[Title/Abstract]

46. Intervention[Title/Abstract]

47. (40 to 46, OR)

Emergency Department

48. Emergency Service, Hospital[MeSH Terms]

49. "Emergency Service, Hospital"[Title/Abstract]

50. “Emergency Department”[Title/Abstract]

51. “Emergency Services, Hospital”[Title/Abstract]

52. “Hospital Service, Emergency”[Title/Abstract]

53. “Hospital Emergency Services”[Title/Abstract]

54. “Services, Hospital Emergency”[Title/Abstract]

55. “Emergency Hospital Service”[Title/Abstract]

56. “Emergency Hospital Services”[Title/Abstract]

57. “Hospital Services, Emergency”[Title/Abstract]

58. “Emergencies, Hospital Service”[Title/Abstract]

59. “Service, Emergency Hospital”[Title/Abstract]

60. “Services, Emergency Hospital”[Title/Abstract]

61. “Hospital Service Emergency”[Title/Abstract]

62. “Emergency, Hospital Service”[Title/Abstract]

63. “Hospital Emergency Service”[Title/Abstract]

64. “Hospital Service Emergencies”[Title/Abstract]

65. “Service Emergencies, Hospital”[Title/Abstract]

66. “Service Emergency, Hospital”[Title/Abstract]

67. “Emergency Units”[Title/Abstract]

68. “Emergency Unit”[Title/Abstract]

69. “Unit, Emergency”[Title/Abstract]

70. “Units, Emergency”[Title/Abstract]

71. “Service, Hospital Emergency”[Title/Abstract]

72. “Emergency Ward”[Title/Abstract]

73. “Departments, Emergency”[Title/Abstract]

74. “Emergency Wards”[Title/Abstract]

75. “Ward, Emergency”[Title/Abstract]

76. “Wards, Emergency”[Title/Abstract]

77. “Emergency Departments”[Title/Abstract]

78. “Department, Emergency”[Title/Abstract]

79. “Emergency Department”[Title/Abstract]

80. “Rooms, Emergency”[Title/Abstract]

81. “Emergency Room”[Title/Abstract]

82. “Emergency Rooms”[Title/Abstract]

83. “Room, Emergency”[Title/Abstract]

84. emergency medical services[MeSH Terms]

85. "Emergency Medical Services"[Title/Abstract]

86. (48 to 85, OR)

87. (39 AND 47 AND 86)

## Abbreviations

ED: Emergency department
BCIs: Brief contact interventions
RCTs: Randomized clinical trials
PROSPERO: International Prospective Register of Systematic Reviews
MesH: Medical Subject Headings
PICO: Population, Intervention, Comparator and Outcomes
ICD: International Statistical Classification of Diseases and Related Health Problems
EMBASE: Excerpta Medica dataBASE
DARE: Database of Abstracts of Reviews of Effects
CINAHL: Cumulative Index to Nursing and Allied Health Literature
BVS: Virtual Health Library
OATD: Open Access Theses and Dissertations
ROBINS-I: Risk Of Bias In Non-randomized Studies - of Interventions
ROB 2: Risk of Bias 2
GRADE: Grading of Recommendations, Assessment, Development and Evaluation
CI: Confidence interval
