# Supplementary material for: Effects of psychosocial interventions among people cared for in emergency departments after a suicide attempt: a systematic review protocol
Source: Syst Rev. 2021 Mar 5;10:68. doi: 10.1186/s13643-021-01609-5 (PMC7992994; doi:10.1186/s13643-021-01609-5)
Supplement: Supplementary file 2 — Additional file 2. Cochrane data collection form. [file 13643_2021_1609_MOESM2_ESM.pdf]

Data collection form

[Additional file 2]

Intervention review – RCTs and non-RCTs (Cohort and case-control studies)

Instrument adapted from Data collection form – RCTs and non-RCTs, Cochrane Collaboration. Adapted by Ana Paula Coutinho da Silva and reviewed by Inês Areal Rothes.

Reference: The Cochrane Collaboration. Developmental, Psychosocial and Learning Problems. Data collection form for intervention reviews for RCTs and non-RCTs - template. https://dplp.cochrane.org/data-extraction-forms. Accessed September 2020.

Notes on using data extraction form:

- Be consistent in the order and style you use to describe the information for each report.
- Record any missing information as unclear or not described, to make it clear that the information was not found in the study report(s), not that you forgot to extract it.
- Include any instructions and decision rules on the data collection form, or in an accompanying document. It is important to practice using the form and give training to any other authors using the form.

Identification

Report ID	
Report ID of other reports of this study	

General Information

Study title	
Authors	
Year of publication of the study	
Journal	
Country in which the study was developed	
Study author contact details	
Type of publication	
Abstract	

Notes	

Study eligibility

Study Characteristics	Eligibility criteria<br><br>*(Insert inclusion criteria for each characteristic as defined in the Protocol)*	Excel Code	Eligibility criteria met?<br><br>Y N U
I. Type of study	Randomised Controlled Trial	(1)	☐ ☐ ☐<br>Yes: (1) to (4)<br>No: (5)<br>Unclear: (0)
	Quasi-randomised Controlled Trial	(2)	
	Case-control study	(3)	
	Cohort study	(4)	
	None of these	(5)	
	Unclear	(0)	
II. Participants	Suicide attempt	(1)	☐ ☐ ☐<br>Yes: (1) to (5)<br>No: (6)<br>Unclear: (0)
	Self-harm	(2)	
	Suicidal behavior	(3)	
	Self-poisoning	(4)	
	Self-Injury	(5)	
	This is not suicidal behavior	(6)	
	Unclear	(0)	
	Others ________________________		
III. Types of intervention	Intensive follow-up with scheduled visits	(1)	☐ ☐ ☐<br>Yes: (1) to (10)<br>No: (11) and (12)<br>Unclear: (0)
	Home visit to patients who did not keep outpatient appointment	(2)	
	Assertive intervention with outreach consultations	(3)	
	Brief intervention and contact	(4)	
	Letter or postcard	(5)	

	Telephone	(6)	
	Composite of letter/postcard and telephone	(7)	
	Psychotherapy group	(8)	
	Psychotherapy individual	(9)	
	Psychotherapy individual and group	(10)	
	Not applicable (the studies do not test an intervention)	(11)	
	Not applicable (pharmacological intervention)	(12)	
	Unclear (an intervention was tested but it is not clear which)	(0)	
	Others ________________________________		
IV. Types of comparison	Usual treatment	(1)	It is not an exclusion criterion.
	Usual improved treatment	(2)	
	Not applicable (the studies do not test an intervention)	(3)	
	Not applicable (pharmacological intervention)	(4)	
	Unclear	(0)	
	Others ________________________________		
V. Context	Emergency Department	(1)	☐ ☐ ☐<br>Yes: (1)<br>No: (2) or any other health scenario.<br>Unclear: (0)
	Psychiatric Emergency Department	(2)	
	Unclear	(0)	
	Others ________________________________		
VI. Types of outcome measures	Repeated suicide attempt	(1)	It is not an exclusion criterion.
	Death by suicide	(2)	
	Adherence to referral for health follow-up	(3)	
	Suicidal ideation	(4)	
	Psychological symptoms	(5)	
	Social functioning	(6)	

	Not applicable	(7)	
	Unclear	(0)	
	Others ______________________		

Y = Yes   N = No   U = Unclear

For any characteristic that is not clear the information (U), enter 0 (zero) in the excel field.

INCLUDE ☐ EXCLUDE ☐ UNCLEAR ☐

To include, answered "yes" the characteristics: I, II, III, V.

To exclude, he answered "no" to any of the characteristics: I, II, III, V.

For Unclear, he replied "unclear" to any of the characteristics I, II, III, V.

In Excel, Include check mark (1)

In Excel, Exclude check (2)

In Excel, Unclear score (0)

1st phase of eligibility (Title and Summary) - Reasons for exclusion:	Codes for Excel:
Study type	(1)
Ineligible population	(2)
Ineligible intervention / Do not test intervention	(3)
Ineligible scenario	(4)

2st phase of eligibility (Full text) - Reasons for exclusion:	Codes for Excel:
Study type	(1)
Ineligible population	(2)
Ineligible intervention / Do not test intervention	(3)
Ineligible scenario	(4)
Self-inflicted injury without suicidal intent	(5)

In case of doubts: Any doubts about filling in contact with the researcher responsible for the email: apaulinh@yahoo.com.br

DO NOT PROCEED IF STUDY EXCLUDED FROM REVIEW

Sources:

Cochrane Collaboration Glossary, 2010. Available from http://www.cochrane.org/training/cochrane-handbook.

Higgins JPT, Green S (editors). Cochrane Handbook for Systematic Reviews of Interventions Version 5.1.0 [updated March 2011]. The Cochrane Collaboration, 2011. Available from www.cochrane-handbook.org.

Last JM (editor), A Dictionary of Epidemiology, 4th Ed. New York: Oxford University Press, 2001.

Schünemann H, Brożek J, Oxman A, editors. GRADE handbook for grading quality of evidence and strength of recommendation. Version 3.2 [updated March 2009]. The GRADE Working Group, 2009. Available from http://www.cc-ims.net/gradepro.
